# Graphene: The Missing Piece for Cancer Diagnosis?

**DOI:** 10.3390/s16010137

**Published:** 2016-01-21

**Authors:** Sandra M. A. Cruz, André F. Girão, Gil Gonçalves, Paula A. A. P. Marques

**Affiliations:** 1 Coimbra Chemistry Center, Department of Chemistry, University of Coimbra, Coimbra 3004-535, Portugal; xcruz@ci.uc.pt; 2 Nanoengineering Research Group, TEMA, Department of Mechanical Engineering, University of Aveiro, Aveiro 3810-193, Portugal; andrefgirao@ua.pt (A.F.G.); ggoncalves@ua.pt (G.G.)

**Keywords:** graphene, biosensors, cancer biomarkers, cancer cells

## Abstract

This paper reviews recent advances in graphene-based biosensors development in order to obtain smaller and more portable devices with better performance for earlier cancer detection. In fact, the potential of Graphene for sensitive detection and chemical/biological free-label applications results from its exceptional physicochemical properties such as high electrical and thermal conductivity, aspect-ratio, optical transparency and remarkable mechanical and chemical stability. Herein we start by providing a general overview of the types of graphene and its derivatives, briefly describing the synthesis procedure and main properties. It follows the reference to different routes to engineer the graphene surface for sensing applications with organic biomolecules and nanoparticles for the development of advanced biosensing platforms able to detect/quantify the characteristic cancer biomolecules in biological fluids or overexpressed on cancerous cells surface with elevated sensitivity, selectivity and stability. We then describe the application of graphene in optical imaging methods such as photoluminescence and Raman imaging, electrochemical sensors for enzymatic biosensing, DNA sensing, and immunosensing. The bioquantification of cancer biomarkers and cells is finally discussed, particularly electrochemical methods such as voltammetry and amperometry which are generally adopted transducing techniques for the development of graphene based sensors for biosensing due to their simplicity, high sensitivity and low-cost. To close, we discuss the major challenges that graphene based biosensors must overcome in order to reach the necessary standards for the early detection of cancer biomarkers by providing reliable information about the patient disease stage.

## 1. Introduction

Cancer is considered to be one of the leading causes of health loss worldwide and both its early diagnosis and efficient treatment still present challenges to overcome, despite great progress made in the past few decades [1,2]. The current standard controlling of cancer patients involves stage determination (by imaging or biopsies), chemo/radiation therapy, and/or surgical resection. Different molecular imaging techniques, including optical imaging by fluorescence and Raman spectroscopy have furthered our understanding of cancer initiation, progression, and metastasis [3]. Molecular imaging is also a useful tool to monitor *in vivo* biochemical events that can provide information about cancer diagnosis and therapeutic response [4]. 

The aspired goal in an accurate and early-stage diagnosis of cancer is based on the detection and quantification of reliable cancer biomarkers by cost-effective and less invasive methods. Researchers believe that early diagnosis of diseases with minimal cost and time-consumption may become achievable due to recent advances in the development of biosensors. These devices use biorecognition elements for the selective interaction with an analyte and different types of transducers to obtain the signal readout. The operational characteristics of biosensors have been reported as improving substantially when nanomaterials such as graphene and its derivates are employed. Graphene is aromatic, hydrophobic and chemical inert; moreover, it is biocompatible and has the facility to adsorb biomolecules due to π-π stacking between its hexagonal cells and the carbon rings present in the majority of nano/biomolecules. Owing to its exceptional charge mobility and atomic thickness, graphene have been proposed as the basis to sensitive detection and chemical/biological free-label [5,6]. Furthermore, since graphene is a two-dimension nanostructure, it does not present the geometric constrains observed for other carbon-based devices. Additionally, graphene oxide (GO), the oxidative form of graphene has attracted the attention of scientists because its surface chemistry is highly versatile due to the presence of oxygen groups capable of enabling not only the use of several functionalization approaches but also the preparation of multifunctional nanocomposites, extending the range of the applications of these nanomaterials in biosensores. 

Herein we intent to discuss the most relevant graphene based technology for the development of different types of biosensors skillful to detect characteristic cancer biomolecules in early stages or overexpressed on cancerous cells surface. We summarize the current existing challenges on the cancer biomarker detection and quantification through graphene based biosensors in order to increase the selectivity and sensitivity. Finally, we intent to give a global view about the challenges for future development of various types of graphene based biosensors for cancer detection.

## 2. Graphene and Its Derivatives

Theoretically, graphene sheets are perfect 2D single crystal formed by sp^2^-hybridized carbon bonds in aromatic structure. Although at the moment several synthetic methodologies allow to obtain this material, most of them are really far from provide the theoretical structure, producing instead graphene sheets with different chemical or/and physical defects. The most common defects observed on “graphene” are: sheets with different numbers of atomic layers that can go from monolayer until multilayer; the atomic flatness is not real because the sheets tend to distort due to the presence of structural defects such as lattice missing carbon atoms or inclusion of other foreign atoms, in particular oxygen; and different lateral dimensions of the sheets [7]. These imperfections have a strong influence on the final optical and electrical properties of graphene and in many cases are related with the synthesis method used for its fabrication [8]. In order to clearly and consistently describe the various derivatives of graphene, graphene based materials can be classified according to three major physical-chemical features (number of layers, quantity of oxygen and lateral dimensions) [9]. This standardization of graphene materials offers the possibility to have an effective control of the major factors that influence graphene properties, which is really important for the development of sensors in a reproducible and consistent way.

**Figure 1:**
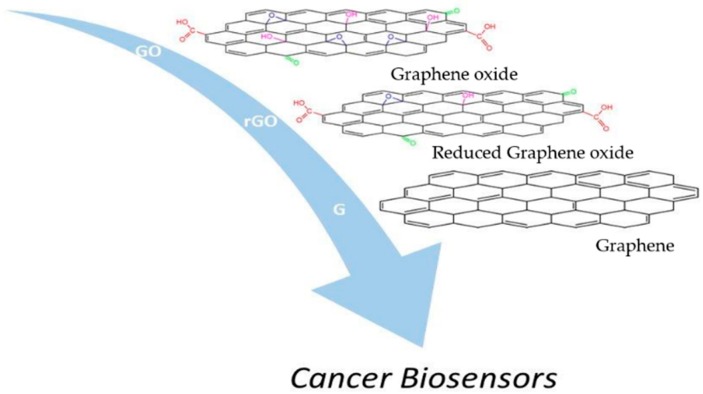
Illustration of graphene derivatives with potential applications on cancer biosensors.

Hereafter we describe the family of graphene and its derivatives (Figure 1), their main properties and preparation methods as well as some methodologies used to surface modify these nanostructures and consequently increase their sensing performance.

### 2.1. Graphene

Chemical vapor deposition (CVD) production of graphene offers the possibility to obtain large sized films that are ideally for the fabrication of electronic sensor devices with high specific detection area and low noise [10]. However, some problems can be found when using this type of methodology since graphene can present some defects and impurities and few-layered domains that really affects the electron mobility and limits the size of device fabrication. Some practical problems associated with the need to transfer the produced graphene sheets to insulating substrates for the sensor development are reported elsewhere [11]. Despite the limitations associated with the this synthesis method, recently a chemiresistive sensor based on a single layer graphene produced by CVD was reported to exhibit excellent selectivity toward eleven chemically distinct compounds and nine mono-substituted benzene compounds with an accuracy of 96% and 92%, respectively [12]. The sensor used throughout each experiment did not have any surface modifications to enhance selectivity; and therefore, the observed responses were inherent to the material and device itself. These results are very a promising for future development of multiarrays graphene based sensor. 

The synthesis of large graphene sheets is also possible through the epitaxial growth on the basal plane of single-crystal silicon carbide. The big advantage of this approach is that graphene films formed at silicon carbide (SiC) surface can be directly used for sensor development since the substrate is a good insulator and the strong interaction between graphene and SiC creates a graphene bandgap to 0.26 eV, which is ideal for sensors whose detection is based in induced field effect [13]. However graphene growth at SiC surface shows some typical defects such as substrate induced corrugations and rotational disorder between layers resulting in films with heterogeneous thickness [14].

### 2.2. Graphene Oxide

The wet chemical exfoliation of graphite is a very common adopted approach for the synthesis of GO in large scale and at low cost. GO is an electrical insulator, chemically heterogeneous with high amount of oxygen groups linked to the carbon lattice forming defect regions with sp^3^ C-C bonds [15]. GO has a huge potential for the development of optical sensors due to its particular properties for optical sensing: photoluminescence over a broad range of wavelengths and highly efficiency as a fluorescence quencher [16]. Furthermore, GO can also be chemically engineered in order the tune its optoelectronic properties, from insulator to semiconductor or semi-metal, by applying chemical, thermal or electrochemical reduction methods [17]. The resultant material, reduced graphene oxide (rGO), is characterized to have high degree of defects on carbon sp^2^ lattice and residual oxygen contents (dependent of the reducing method applied). Nevertheless, rGO is considered the most promising material for electrochemical sensors, since it is electrochemically more active than any other graphene family member [18]. Furthermore, rGO has an interesting potential for the development of electronic sensing due to its capability to acquire high on-off ratio dependent of applied voltage [19]. The pioneer work on the development of electrical biosensors using rGO was made by Mohanty and Berry [20]. They demonstrated the viability of using rGO as sensitive building block for bioelectronics at both microbial and molecular levels: as either a single bacterium resolution interfacial device, a label free reversible DNA detector or a polarity-specific molecular transistor for protein/DNA adsorption. After that many other works have been published using rGO for the development of electronic sensors [21].

### 2.3. Nano-Graphene and Nano-Graphene Oxide

Graphene quantum dots (GQDs), also referred as Nano-graphene (NG) are nanometer-sized fragments with distinct properties from graphene, which makes them interesting candidates for a whole range of new applications. The methods to synthesize NG can be classified into two types: the top-down and bottom-up methods [22] being the top-down routes that possess the advantages of having abundant raw materials, large scale production and simple operation the most applied.

The top-down approaches include hydrothermal [23], electrochemical [24], solvothermal and microwave-assisted cutting, between others. Recently, Li *et al*. [25] reported these methodologies in a systematic review. The bottom-up methodologies include mainly stepwise organic synthesis using organic molecules like polycyclic aromatic hydrocarbons [26] and also cage opening of fullerenes [27]. Indeed, it is significant to synthesize stable colloidal NG with customized and controllable size in order to obtain tunable fluorescence NG [28] which diameter distribution ranges from 3 nm to 20 nm [29].

Nano-graphene oxide (NGO) is a new class of carbon based materials that was proposed for biomedical applications due to its small size, intrinsic optical properties, large specific surface area, and easy to functionalize [30]. The preparation methods for NGO are mainly based on top-down methodologies [31]. The high degree of oxidation of the obtained GO grants this material with a high hydrophilic character which allows to obtain very stable aqueous colloidal suspensions and able for easily functionalization [32].

## 3. Engineering Graphene Surface for Sensing Applications

One very simple way to induce further sensing capabilities to graphene is by changing its physical-chemical structure. The changes required to obtain new graphene features can be made by the chemical doping of its structure with different foreign atoms that promote changes in electrical, electrochemical and optical properties. The most typical methodologies for atomic modifications of graphene surface includes oxygenation, hydrogenation and fluorination by using different synthetic approaches [33]. Another very conventional way is by grafting graphene surface with recognition elements that can act as detection targets promoting specific interactions with an analyte. Many parameters should be considered during the engineering of the sensing surface of graphene: the specific sensing target, the sensing mechanism, performance (detection limit and dynamic range), reproducibility, cost and manufacturing.

### 3.1. Graphene Functionalization with Small Molecules and Biostructures

There are two main different approaches for the surface functionalization of graphene based materials: covalent and noncovalent. The covalent functionalization is very straightforward when using as a precursor GO sheets. The presence of oxygen functional groups like carboxylic or hydroxyl at GO surface enables the establishment of covalent bonds with other molecules, polymers, biological structures and nanoparticles. The range of chemical mechanism available to design new covalent bonds between graphene and other organic entities is very broaden following the basic principles of organic chemistry [34]. The organic covalent reactions of graphene include two general routes: the formation of covalent bonds between free radicals or dienophiles and sp^2^ hybridized carbon bonds of graphene and/or the formation of covalent bonds (usually via amide or ester formation) between the functional molecule and the oxygen groups at graphene surface [35]. For example, oligothiophenes can be grafted on GO sheets through covalent amide bonds resulting in an improved absorption in the whole spectral region and an efficient quenching of photoluminescence able to exhibit enhanced nonlinear optical and optical limiting properties that can be explored for the development of new optoelectronic sensors [36]. Many other examples are reported on the literature describe the covalent modification of GO at molecular level via charge transfer between electron donor and electron acceptor molecules allowing significant changes in the electronic structure of graphene [37]. In general, covalent functionalization of GO leads to better performance than noncovalent functionalization due to a more effectively improved energy or electron transfer from the functional moiety to the graphene nanomaterial. Non-covalent bonds between GO and others structures or molecules are mainly governed by electrostatic interactions or hydrogen bonding due to the high electronegativity character of its surface [38]. This feature provides a very hydrophilic nature to GO, which was successfully exploited for the development of humidity sensors with unprecedented response speed [39]. Furthermore, GO was also explored as platform for the establishment of non-covalent bonding with DNA or proteins that allows the biorecognition events during biosensing [40].

In case of graphene, although covalent strategies can be more efficient and stable they request dramatically changes on the structure by the disruption of the C=C bonds leading to the alteration of its characteristic electronic properties. For this reason, non-covalent functionalization of graphene is considered a simple and versatile method, offering the opportunity to attach different functionalities while simultaneously maintaining the integrity of the C-C sp^2^-hybridized carbon network. That fact offers a number of unique physicochemical properties that are desirable and advantageous for sensing applications. Pristine graphene possesses an extensive aromatic surface that allows the molecular interaction via π-π stacking and due to the atomic thickness the electric, mechanic and optical properties are highly sensitive to adsorbed molecules. Recently, the optoelectronic properties of graphene were explored as a biosensor for chemically specific label-free detection of proteins [41]. The electromagnetic fields of graphene infrared (IR) plasmons display unprecedented spatial confinement which allows its using for enhanced light-matter interactions and integrated mid-IR photonics. The large mismatch between mid-IR wavelengths and biomolecules dimensions causing weak vibrational absorption signals is overcome by exploiting the presence of graphene plasmons near to biomolecules. Tunable plasmon resonance of nanostructured grapheme allows biomolecules high sensitivity by the extraction of its complex refractive index and the identification of vibrational fingerprints [41]. For example, an interesting work showed that the functionalization of graphene with thionine by π-π stacking at the surface of glass carbon electrode allows the development of electrochemical sensor that can selectively recognize polycyclic aromatic hydrocarbons with high sensitivity and low detection limit [42]. 

Reduced Graphene Oxide as explained before results from the partial recovery of aromatic structure through the gradual reduction of GO, showing an hybrid structure between sp^2^/sp^3^ carbon regions on a global network [38]. rGO offers a large panoply of non-covalent bonding possibilities, being the degree of oxidation of those materials that balance the preferential chemical bonds that it can establish: π-π stacking, electrostatic interactions or hydrogen bonding. This material has the particular ability for the establishment of oxidation/reduction reactions with different molecules or structures. The electrical properties of rGO have been previously shown to be very sensitive to surface adsorbates, thus making rGO a very promising platform for highly sensitive electrochemical sensors [43], molecular sensors [44] and gas sensors [45]. Despite all advantages of rGO for sensing applications, rGO-based gas sensors have poor selectivity because different gas molecules could be adsorbed on its surface and promote changes on the conductivity. Recently, the functionalization of rGO sheets through π–π stacking with tetra-α-iso-pentyloxyphthalocyanine showed an improved sensing performance to NH_3_ gases at room temperature in comparison to that of pure rGO [46]. The enhanced sensing properties are attributed to the synergistic effect in rGO hybrids, with strong electron transfer interaction, superior electrical conductivity, and gas adsorption activity. Many other examples of the increase selectivity of rGO based materials through non-covalent interactions can be found in literature [21].

### 3.2. Graphene Nanoparticles Hybrid Materials

The closest integration of nanoparticles on graphene based materials surface are of a particular interest because it offer the possibility to obtain the individual properties of each element in a single material and at same time exhibit additional synergistic effects that can be very important on sensor development through the development of new sensing mechanism or increasing the sensitivity and selectivity of the system. The functionalization of graphene, GO and rGO with nanoparticles can occur by both covalent and noncovalent interactions using many different synthetic routes: growing the nanoparticles on their surface (in situ) or by attaching pre-synthesized nanoparticles to the graphene surface (*ex situ*) [47]. However, GO and rGO are preferential substrates for the nucleation and growth of inorganic nanoparticles due to the high level of oxygen function groups and structural defects. Several hybrid graphene based materials have been described on literature using gold, silver, titanium dioxide, and iron oxide and nickel nanoparticles for sensor applications [47]. The majority of graphene nanoparticle hybrid sensors developed are based on the electronic, electrochemical, and optical sensing mechanisms [48]. Nanoparticles can be useful for sensing system in many different ways: enhance surface area for detection, preserve the electrical properties of graphene by conjugating the probe, immobilization of biomolecules, catalyse electrochemical reactions, act as a reactant, double-quenching and dual enhancement of Raman signals via chemical and electromagnetic interactions. A recent review describes in detail the current progress that has been made in the development and application of graphene–nanoparticle hybrid sensors [49]. The greatest challenge that this technology faces at the moment consists on the fabrication of graphene–nanoparticle hybrid sensors able to produce sensing results in a reproducible way.

## 4. Biosensing Cancer Biomolecules or Cells

In a near future, biosensors are expected to detect with high sensitivity and selectivity a wide range of cancer biomarkers present in body fluids or overexpressed on cancer cells surface in order to diagnosis and monitoring the evolution of the disease during the treatment. In this way, biosensing systems employing the properties of graphene derivatives for cancer detection have found their way into a wide variety of strategies capable of improving the sensing efficiency of cancer biomarkers. In the following sections, we describe the application of graphene in optical imaging methods such as photoluminescence and Raman imaging, electrochemical sensors for enzymatic biosensing, DNA sensing, and immunosensing. 

The changes that occur in the DNA sequence of the genomes of cancer cells are the reason of cancers arising [50]. The profound metabolic remodeling of cancer cells, including mitochondrial rearrangement, is an indirect response to cell survival or proliferation, being controlled by specific cell signaling [51]. The monitoring of molecular and physical events in live cells by visualization represent a key approach to understanding cell biology and have a profound influence on biomedical sciences progress. Biomedical sensors that can detect selective signals for biological molecules or physical variables in live cells with spatiotemporal resolution are a constant need. Fluorescent spectroscopic techniques offer sensing for intracellular signaling and analysis due to their non-invasiveness and high sensitivity [52]. These optical features are of particular interest in the detection of cancer cells because they allow a sub-cellular resolution and therefore the capability of identifying tumor phenotypes at their very early stages [53]. Although the organic dyes and fluorescent proteins are powerful molecular probes, they present limitations in making reliable intracellular measurements because of their poor photobleaching resistance [52]. In addition, their broad emission spectra may hamper practical applications since different fluorophores require multiple excitation wavelengths [54]. 

The possible chemical interactions of this type of powerful molecular probes or steric hindrance with biomolecules may cause biotoxicity or perturbation to the systems being investigated which effect is not conceivable in medical applications [55].

### 4.1. Photoluminescence

The development of nanoparticles with multifunctionalities has been a research focus in the last decade including graphene and its derivatives [56]. The unprecedented characteristics of these nanomaterials lead the researchers to suggest it as a new class of fluorescent probes for biomedical imaging. NG is photostable, non-toxic and easily conjugable [56,57]. NG and NG derivatives can be used as fluorescence probes in photoluminescence imaging. Additionally, photoluminescence studies demonstrate that these carbon fluorescent probes may have two functionalities: specific detection of cancer cells and therapeutic agent (drug/gene vehicle or photodynamic therapy agent), that is present both cancer imaging and therapeutic functionalities [58,59,60].

**Figure 2:**
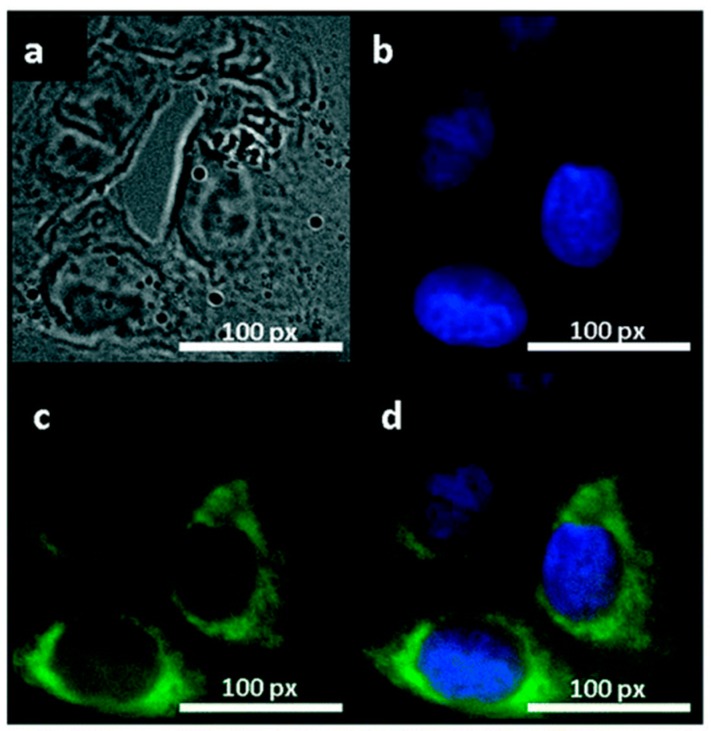
Fluorescent images of human breast cancer cell T47D after incubation with green GQDs for 4 h (**a**) phase contrast picture of T47D cells; (**b**) Individual nucleus stained blue with DAPI; (**c**) Agglomerated green GQDs surrounding each nucleus; (**d**) The overlay high contrast image of nucleolus stained with blue DAPI and GQDs (green) staining. Reproduced with permission [61] Copyright 2012, American Chemical Society.

The high contrast bio-imaging produced by NG in the detection of cancer cells was demonstrated in the work of Peng *et al*. [61] The green NG was used to enhance the contrast visualization of the cells compounds. The nucleus of human breast cancer cell lines T47D were stained with DAPI and show blue color under imaging. After staining, cancer cells were treated with green NG during 4 h of incubation. The obtained images clearly showed the phase contrast image of T47D cells, nucleus stained blue with DAPI and high contrast fluorescent image of green NG around each nucleus (Figure 2).

The photoluminescence of GO was first used for cellular imaging by Dai *et al*. [62] NGO was pegylated and conjugated covalently with a B-cell specific antibody Rituxan (anti-CD20) for selective binding to B-cell lymphoma cells. Since NGO showed luminescence in the visible and near infrared (NIR) regions the optical identification of cancer cells was possible. Additionally, NGO showed a dual functionality as drug carrier for Doxorubicin (DOX) and contrast agent for cancer cell imaging. 

*In vivo* studies using NG prepared by a simple hydrothermal method with polythiophenes as the precursors indicated that NG can act as a multifunctional nanoplatform for the simultaneous imaging and highly efficient in photodynamic therapy (PDT) of cancer [63]. Tumor-targeting ligands such as folic acid (FA) [64] and hyaluronic acid (HAu) [58] could be easily coupled onto NG for tumor targeting. Wang *et al.* [64] developed a study where NG-FA was incubated with three different cell types that express FA receptor at different levels to demonstrate that FA preserves its binding activity to FA receptor even after its conjugation with NG. It was possible to observe that the fluorescence of NG in the human cervical carcinoma cell line HeLa is considerably stronger than in the adenocarcinomic human alveolar basal epithelial cell line and human embryonic kidney cell line HEK293A. In this study, the authors also proved that the green fluorescence arises from the internalization of NG-FA. Moreover, the results reveal the specific internalization of NG-FA by HeLa cells, consistent with the fact that HeLa cells overexpress FA receptor while A549 and HEK239A express FA receptor at a low level, indicating that NG-FA is internalized via endocytosis induce by FA receptor. 

**Figure 3:**
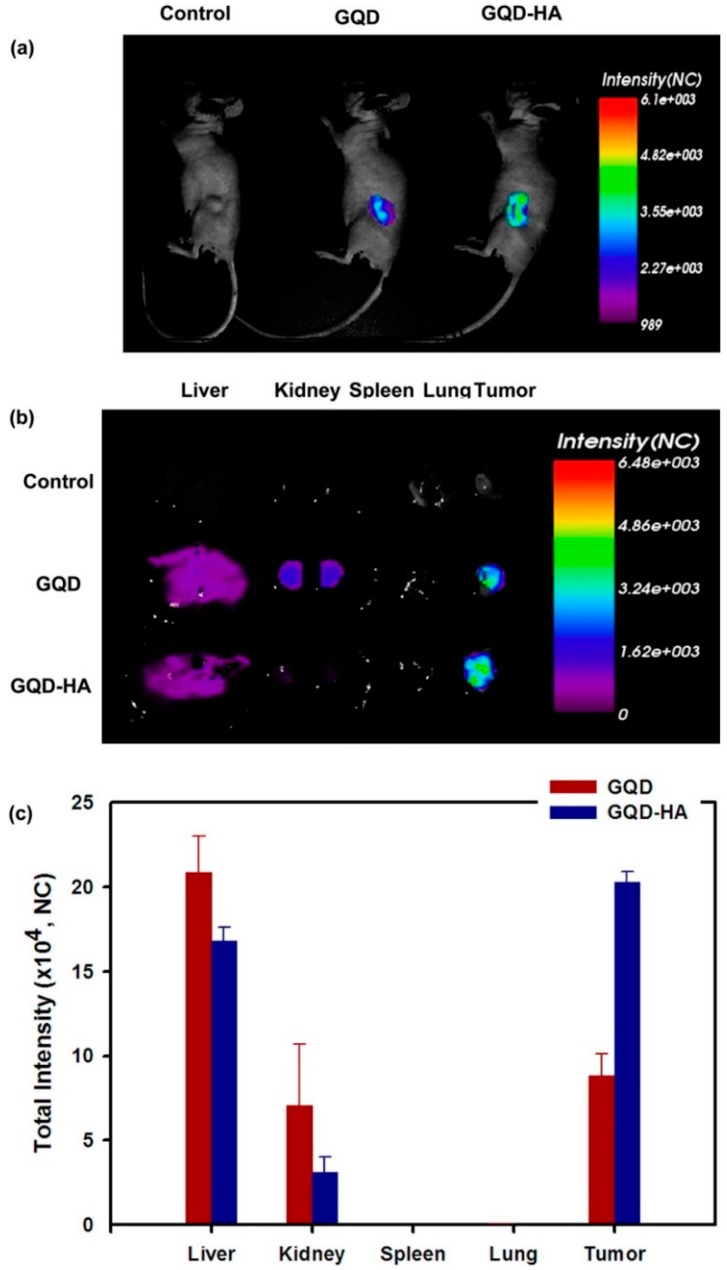
(**a**) *In vivo* fluorescence images of GQD-HA in mice after tail vein injection; (**b**) Ex vivo images of liver, kidney, spleen, heart, and tumor after dissection; (**c**) Normalized intensity from dissected organs .Reprinted with permission from [58]. Copyright (2013) American Chemical Society.

The hyaluronic acid was conjugated with NG by Abdullah-Al-Nahain *et al*. [58] The nanographene-hyaluronic acid (NG-HA) nanocomposite was tested on both mammalian cell lines MDCK (CD44 negative) and A549 of lungcarcinoma with CD44 overexpressed. The CD44 antigen is expressed in a large number of mammalian cells, it is a receptor for hyaluronic acid and participate in a wide variety of cellular functions, including tumor metastasis. Using findings from the confocal and cellular uptake investigation, it is clearly indicated that a larger amount of NG-HA was taken up by A549 cancer cells. On contrary, small amounts of non-targeted NG were allowed to enter into both types of cells, in which due to the presence of HA more NG-HA entered the cell cytoplasm via receptor-mediated endocytosis-based target delivery. The *in vivo* biodistribution and tumor specific delivery of NG-HA were also studied by imaging fluorescence. To investigate the efficient delivery of NG, the authors have used balb/c mice with 6 weeks of age. The tumors were grown, during 10 days (volume of 100mm3) on the back of the mice using A549 cells. to tumor tissue and its biodistribution, NG-HA was injected intravenously (10 mg/kg of body weight) through the tail vein. After 2 h, the mice with tumors were sacrificed and their organs dissected. The strong fluorescence from the tumors indicates the targeted accumulation of NG-HA to the tumor site (Figure 3). The biodistribution profiles of NG-HA showed that this nanocomposite was significantly accumulate in the tumor tissue due to the large number of leaky blood vessels surrounding the tumor. NG-HA was also accumulated in great quantities in liver and kidney tissue (Figure 3): blood circulation pass through liver and NG was uptake by the reticuloendothelial system and the accumulation of the kidney indicates a rapid excretion of this nanocomposite. Once again, the fluorescent NG-HA was also used to deliver DOX as treatment to cancer cells. The results showed that 60% was released within 12 h under the studied conditions. Moreover, it was found that almost all drugs were released within 48 h from the NG-HA matrix.

In addition to tumor cell detection and recognition, NG can also be useful to monitor biochemical pathways, such as apoptosis, the programmed cell death that is a way for multicellular animal dispose of unwanted and damaged cells. The improper regulation of apoptosis has been associated to cancer, neurodegenerative and cardiovascular diseases [65,66]. Nonetheless, how the abnormal cell apoptosis begins and progress *in vivo* is not yet completely understood. The understanding of the various forms of apoptosis could be the key to unlocking the diagnosis and therapy of the related diseases. Numerous methods have been developed for imaging apoptotic cells *in vitro* [67,68], however there are few methods available for imaging apoptotic cells in live animals. 

Recently, Roy *et al.* developed a novel method using NG modified with Annexin V antibody (AbA5) to form (AbA5)-modified NG (AbA5-NG) enabling to label apoptotic cells in live zebrafish (Danio rerio) [69]. Zebrafish showed bright red photoluminescence in the presence of apoptotic cells. The fluorescent images of normal cells (MCF-10A cells) and cancer cells (HeLa and MCF-7 cells) after treatment with NG (2 mg·mL^−1^) were separately recorded. It was possible to observe that the photoluminescent spots were distributed around the cell membranes and cytoplasm suggesting that NG penetrated into the cells via receptor-mediated and/or nonreceptor-mediated endocytosis. This study opens a novel opportunity to visualize the initiation and progression of apoptosis which might help in understanding the apoptotic mechanism better which is of major importance in diagnosis and treatment of cancer.

### 4.2. Surface Enhanced Raman Scattering

In the past few years the first reports of the feasibility of *in vivo* imaging of cancer with biocompatible surface-enhanced Raman scattering (SERS) probes have emerged although its application is traditionally *in vitro*. Fluorescence imaging is the most common bioimaging technique [57,70,71] for the reasons mentioned in the previous sub-section but various intrinsic limitations including photobleaching due to unstable fluorescent dyes, autofluorescence from the biological samples, or limited multispectral detection owing to the spectral overlap between broadband fluorescence spectra hinders the further applications of fluorescence microscopy imaging [72,73]. In contrast, Raman scattering provides narrow spectral bandwidth and is resistant to photobleaching and autofluorescence and suitable for long-term monitoring of cellular processes [74,75]. The low Raman scattering of an analyte, especially of biological nature, can be strongly enhanced by locating it near the surface of a noble metal nanostructure. This process known as surface-enhanced Raman scattering (SERS) makes that the amplification of the received signal overcomes the limitation of the intrinsically low Raman scattering efficiency of Raman spectroscopy [71]. SERS is capable of enhancing the Raman signals of analytes located near the surfaces of noble metal nanostructures by up to 10 orders of magnitude [70,76]. This allows the probing and imaging of cells with high sensitivity using SERS labels [77,78,79]. 

Graphene oxide is an excellent candidate for biomedical imaging due to its inherent fluorescence and Raman scattering activity [71]. GO and metal nanoparticle nanocomposites have also been used for diverse SERS applications in cellular imaging. Gold and silver nanoparticles are the most common used in SERS technique and for such reason are strong candidates to perform GO/metal nanocomposites. The presence of metallic nanoparticles enhances Raman characteristic bands, moreover, GO in biological media acts as a fluorescence quencher. These features indicate that GO/metal nanocomposites with specific binders may act as signaling when inside the cancer cells. Hereafter the potentialities of graphene nanocomposites in the detection and, in some cases, therapeutic agent using SERS technique will be discussed.

**Figure 4:**
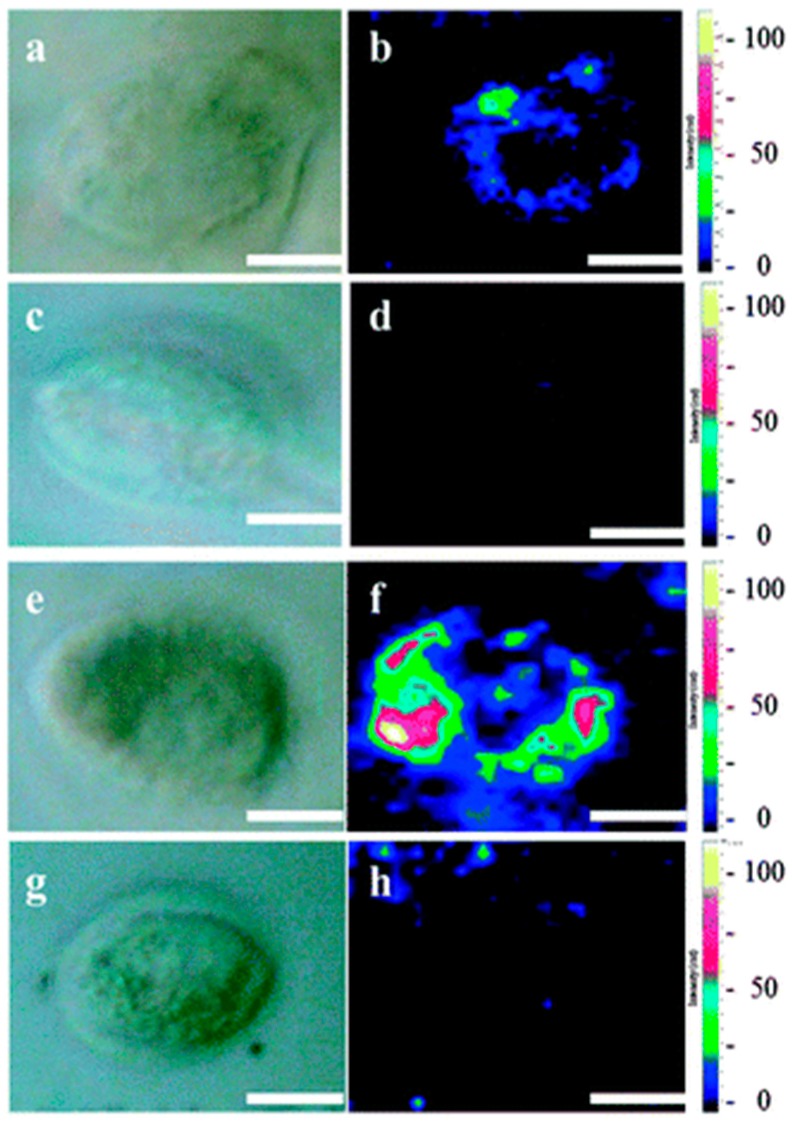
The cellular uptake mechanism investigations of GO and Au/GO hybrids. Hela 229 cells are incubated for 2 h with GO (**a**–**d**) or Au/GO hybrids (**e**–**h**) at 37 °C (**a**, **b**, **e** and **f**) and 4 °C (**c**, **d**, **g** and **h**), respectively. The left panels are optical images while the right panels are the corresponding Raman images (scale bar: 5 mm). Reproduced with permission [80]. Copyright 2012, Royal Society of Chemistry.

GO decorated with Au nanoparticles were internalized into individual cells to provide localized sensing at the subcellular level [80]. The sensitivity was remarkably improved and the acquisition time was effectively shortened as a result of the SERS effect. The cellular uptake mechanism of GO was also investigated. Hella 229 cells were incubated in a medium containing either GO or GO/Au nanocomposites at 4 °C and 37 °C for 2 h respectively. It could be seen that different parts within the cells exhibited different Raman intensities. In the Raman images of the cells (Figure 4), the dark regions correspond to the cell nucleus. The authors concluded that neither GO nor GO/Au went through the nucleus membrane and thus they mainly reside in cytoplasm.

The study of the mechanism of the cellular uptake was also performed by Huang *et al*. [81], using GO loaded with Au nanoparticles as SERS substrate. In this work, once again it was proven the importance of the graphene nanocomposite in the detection of cancer cells besides its contribution on the knowledge about the cellular mechanism uptake. To understand the cell internalization process of the GO/Au, SERS spectra of GO/Au incubated with Ca Ski cells for 1, 2, 4, 6, 8, and 12 h were measured. The strongest SERS signals were recorded after 6 h of incubation. After that, the SERS signal weakened, and was just above the noise level at 12 h of incubation. These results may indicate that, under the present experiments conditions, the amount of GO/Au taken up by Ca Ski cells increased significantly at an incubation time of 4 h, and reached a maximum at 6 h, being the Au nanoparticles released from the GO sheet in the cell environment, after this incubation time.

Manikandan *et al*. have proposed several SERS substrates based on the nucleation of gold nanohexagons (Au) on graphene nanosheets [82]. These were applied to enhance Raman scattering and differentiate human breast normal cancer cells and cancer stem cells. These SERS substrates led to 5.4 fold increase the detection of breast cancer cells and 4.8 fold of sensitivity for detecting breast cancer stem cells. This study demonstrates the potential of graphene to differentiate between normal cells, cancer cells and cancer stem cells by SERS which is considered as the primary tumor focus. 

A gold capture substrate modified with linker molecules and antibodies, covered by a graphene monolayer, was developed by Krasnoslobodtsev *et al*. [83]. In this work, pancreatic cancer cells presented the mucin protein (MUC4) overexpressed, which was used as a biomarker in serum-based assay for early diagnosis of such type of tumor. As MUC4 was not detected in serum by the conventional bioassays used in clinical settings as enzyme linked immunosorbent assay (ELISA) and radioimmunoassay (RIA), the authors prepared a SERS substrate for MUC4 sandwich immunoassay with a graphene protective monolayer. This SERS-based assay showed the ability to correctly detect and quantify low levels of MUC4, leading to an accurate identification of the cancer prostate patients.Silver nanoparticles have been considered as a superior SERS-active substrate with surface plasmon frequencies from the UV to the near-infrared region changing the ratio of its shell thickness with a core size [84]. A SERS-active nanostructure was prepared by encapsulating Ag nanoshell (AgNS) with GO denoted by GONS [85]. In this work, MCF-7 (human breast adenocarcinoma cell line) cells were treated with GONS. Before the treatment with GONS nanoprobes, the cells had no detectable signals; however, strong SERS signals clearly appeared in the cells that subjected to the treatment. The internalization of this type of nanoprobes shows a higher accumulation of GONS on the cell membrane, although a few were internalized into the cytoplasm. Thirty four different cells were analyzed by Raman spectroscopy and SERS signals of GONS nanoprobes were observed from 82% of the cells which indicates that this graphene-based system is an ultrasensitive nanoprobe for imaging of cancer cells.

Based on the natural high affinity of folate for the folate receptor protein (FR), which is commonly expressed on the surface of many human cancers, folate conjugates bind to the FR and causes the cellular uptake via endocytosis. The conjugation of the specific binding of folic acid (FA) and graphene-metal nanoparticles improve the targeted detection of cancer cells. Liu *et al.* [70] prepared a SERS label based on GO/Ag with FA conjugated covalently. FR-positive HeLa (human cervical cancer) cells and FR-negative A549 (adenocarcinomic human alveolar basal epithelial) were incubated with GO/Ag-FA. Though both types of cells are carcinomic, the binding specificity of FA results in intense Raman signals on imaging of FR-positive cancer cells. A similar study was made by Hu and co-workers [86] by using GO/Ag-FA nanocomposite to distinguish FRs over-expressed cancer cells from the cells on which the FRs are not over-expressed.

In order to apply SERS in real-world, the nucleosides excreted from normal human urine (n = 40) and from breast cancer patients (n = 40) were measured using a graphene-silver-copper nanoparticles (G/SCNP) as SERS probe [87]. On the basis of the obtained G/SCNPs, the SERS spectra of the nucleosides excreted from normal human and breast cancer patients reveal significant difference in some spectral ranges.

The chemotherapy is the most common cancer treatment but its huge side-effects make it very painful for the patients. It is urgent to promote effective and targeted deliver of drugs used in chemotherapy since they show good results. SERS can contribute to monitor such deliver using multifunctional nanoprobes. GO-based nanoplatform simultaneously loaded with chemical drug and Ag nanoparticles was prepared and employed to study the drug release from GO in living cancer model cells by SERS [59]. In this work, DOX was loaded onto GO by electrostatic interaction and Ag nanoparticles were covalently linked onto GO. Real-time measurement of SERS signals of DOX using the GO loaded with Ag NPs as a SERS-active substrate allowed monitoring the process of the drug release inside the living cell. The SERS results revealed that DOX is initially released from the GO surface inside the lysosomes, then escapes into the cytoplasm, and finally enters the nucleus, while GO, the nanocarrier, remains within the cytoplasm, without entering the nucleus. The DOX release into cancer cells was also monitored by SERS imaging using a redox-responsive GO/Ag nanocarrier [60]. The drug was loaded to the surfaces of GO by disulfide linkages which can be cleaved by glutathione (GSH). Covalent drug loading and GSH-responsive release strategy can reduce the influence of the surface diffusion barriers introduced by multifunctionalization. Since tumor cells generally exhibit a higher concentration of GSH than normal ones, this drug carrier should have potential in the field of tumor therapy. GSH triggered drug release process was studied using HeLa cells as a model of tumor cells with a high GSH level. In the presented nanocarriers, the intrinsic Raman bands of NGO were enhanced by attaching Ag nanoparticles on the NGO surfaces, which were used to do SERS mapping images to express the distribution of nanocarriers inside HeLa cells. In addition, the real-time release dynamics were given by detection of SERS signals of DOX *in situ* inside HeLa cells. With such results it was clearly demonstrated that SERS offers distinct advantages over the widely used fluorescence technique in that it can provide direct information on the carrier and the drug inside the living cells simultaneously, and without dye labeling.

## 5. Bioquantification of Cancer Biomarkers and Cells

A biosensor is defined as an integrated device that is able of provides quantitative or semi-quantitative information about biomolecules or biostructures by using biological recognition element. The main difference relatively of a bioanalytical system is that requires additional processing steps such as addition of reagents in order to be able to quantify the desired analyte. The structure of a sensor is usually constituted by two key elements: the receptor and the transducer. The receptor should guaranty the specific interaction with the proper analytic or many different analytes and the transducer is responsible to convert the perturbation caused on the receptor (input) through the interaction with the analyte into a measurable signal (output).

Hereafter we will discuss particularly electrochemical methods such as voltammetry and amperometry which are generally adopted transducing techniques for the development of graphene based sensors for biosensing due to their simplicity, high sensitivity and low-cost. However sensors based on other detection methods like FRET, photoluminescence, fluorescence, optical and SERS start to arise with very promising results on the biodetection of cancer markers.

### 5.1. Electrochemical Sensors

#### 5.1.1. Immunosensors

Immunoassay has becoming one of the most important tools for clinical diagnosis of cancer. Detection of cancer biomarkers associated with certain tumors in early stage of the disease becomes on important contribute for the successful treatment. Graphene sensors promise to be an innovative technology in next generation electronics on cancer diagnostic applications, due to exceptional electronic properties and extreme surface to volume ratio able to offer greatly enhanced sensitivity for the detection of cancer biomarkers. New graphene-based technology allows the development of portable and rapid diagnostic sensors whit potential to overcome the features of conventional enzyme-linked immunobsorbant assays (ELISAs). Hereafter we describe the potential use of graphene and its derivatives on the development of new electrochemical sensors for detection of specific cancer biomarkers.

##### Carcinoembryonic Antigen

Carcinoembryonic antigen (CEA) is one of the most extensively used tumor markers on early detection of lung, ovarian carcinoma, breast cancers and cystadenocarcinoma. A novel electrochemical immunosensor based on carbon ionic liquid electrode (CILE) modified with AuNPs, rGO and poly(l-Arginine) nanocomposite material as sensitive platform for detection of CEA [88]. The synergistic effects of nanocomposite resultant from the individual properties of each material allow an enhanced capacity of adsorption of CEA antibody and a consequently improvement of the electrochemical responses. At optimized conditions, differential pulse voltammetric responses were proportional to CEA concentration in the range from 0.5 to 200 ng·mL^−1^ with the detection limit as 0.03 ng·mL^−1^. Another work reported the development of a novel electrochemical immunosensor based on the deposition of ultrathin Au-Pt nanowire-decorated thionine/reduced graphene oxide (AuPtNWs/THI/rGO) on the surface of glass carbon electrode (GCE) [89]. The differential pulse voltammetric responses were proportional to CEA concentration in the range from 50 fg·mL^−1^ to 100 ng·mL^−1^ with an detection of 6 fg·mL^−1^. A very simple approach using GO-Thi-Au nanocomposites as sensing platform for the surface modification of GCE was been successfully employed for the fabrication of ultrasensitive electrochemical immunosensor for CEA [90]. The immunosensor showed a linear calibration plot in a wide concentration range from 0.1 fg·mL^−1^ to 1 × 10^9^ fg·mL^−1^ and ultralow detection limit of 0.05 fg·mL^−1^.

Electrochemical immunosensors based on Au electrodes modified with graphene nanocomposites as a sensing platform showed to be also a viable approach for the detention of CEA. Samanman *et al*. reported the Au electrode surface modification by deposition of a thin film of silver and then with a AuNPs-rGO-chitosan nanocomposite prepared by cryogel functionalized with anti-CEA [91]. At optimal conditions, the decrease of the cyclic voltammetry silver peak current was proportional to the CEA concentration over a range of from 1.0 fg to 1.0 ng·mL^−1^ with a detection limit of 0.1 fg·mL^−1^. In another work Au electrode surface was initially modified with rGO-SWCNTs-Chitosan nanocomposite (Chi-rGO-CNTs) followed by electrodeposition with Au and Pt NPs and then functionalized with anti-CEA. The proposed immunosensor showed CEA determination of range 0.1 pg·mL^−1^ to 40 ng·mL^−1^ with a limit of detection down to 30 fg·mL^−1^ [92]. Recently Kumar *et al*. reported for the first time a new graphene paper electrode based on poly(3,4-ethylenedioxythiophene):poly(styrenesulfonate)(PEDOT:PSS)/rGO composite as a promising alternative over the expensive conventional electrodes (ITO, gold and GCE) [93]. rGO PEDOT:PSS film was treated with ethylene glycol in order to enhance conductivity, electrochemical activity and charge transfer kinetics and functionalized through the immobilization of the anti-CEA for electrochemical detection of CEA cancer biomarker. This simple, low-cost, flexible PEDOT:PSS/rGO based biosensor shows the sensitivity of 25.8 µA·ng^−1^·mL·cm^−2^ in the detection linear range of 2–8 ng·mL^−1^. 

Graphene biosensors based on sandwich-type immunosensors methods were also been extensively explored on the detection of CEA. High-affinity antibodies and appropriate labels are usually employed for the amplification of detectable signal [94]. Recent research has looked to develop innovative and powerful novel graphene labels or graphene sensing platforms, controlling and tailoring their properties in a very predictable manner to meet the requirements for increasing the detection level and specificity for cancer biomarker. One of the pioneering work, reported a sandwich luminol electrochemiluminescence (ECL) immunosensor using ZnO nanoparticles (ZnONPs) and glucose oxidase (GOD) decorated rGO as labels and *in situ* generated hydrogen peroxide as coreactant (ZnONPs@rGO-GOD-Ab2) [95]. The sensing platform at GCE electrode surface was obtained by the assembly with a hybrid architecture of AuNPs/rGO able to provide high immobilization sites for antibody (Ab1). After the biorecognition event of CEA the ZnONPs@rGO-GOD-Ab2 labels were able to be immobilized on the electrode surface (GCE/rGO-AuNPs/Ab1) via sandwich immunoreactions. Enhanced sensitivity was obtained by *in situ* generating hydrogen peroxide with glucose oxidase and the catalysis of ZnONPs to the ECL reaction of luminol–H_2_O_2_ system. The rGO-based immunosensor exhibited a detection range of CEA from 10 pg·mL^−1^ to 80 ng·mL^−1^ and a detection limit of 0.0033 ng·mL^−1^. Recently, another work describes the use of rGO on both sensor sensing platform and as a signal amplifying factor for construction of the “sandwich-type” immunosensor with ultrahigh efficiency for CEA detection [96]. Graphene sensing platform was prepared by π-π stacking with 1,5-diaminonaphthalene (DN) an then coated with AuNPs conjugated with antigen CEA (Ab1). Graphene tracer label was also based on graphene/DN composite coated with Ag/Au nanoparticles and conjugated with antigen (Ab2). The biorecognition event occurs after the addition of CEA, by immune reactions of antigens and antibodies, which results in the great current signal owing to the existence of electroactive element Ag/AuNPs. The cyclic voltammetric results showed a linear increased of current with the increasing antigen CEA concentrations in the range of 10 to 1.2 × 10^5^ pg·mL^−1^ and a limit of detection 8 pg·mL^−1^. 

Graphene made by CVD was also explored as electrochemical sensing platform, in the form of high surface area conducting electrodes surfaces for the high sensitive detection of CEA. The graphene sensing surface was modified with magnetic beads (MBs) functionalized with CEA detection antibody (Gr/MBs-Ab1), by applying an external magnetic field. In order to enhance the sensitivity of the sensor, AuNPs were modified with horse radish peroxidase (HRP) and detection antibody (Ab2), to form the conjugate Ab2–AuNPs–HRP [97]. The electrochemical immunosensor analysis was based on the biorecognition event with CEA, using GR/MBs–Ab1/CEA/Ab2–AuNPs–HRP system as a tracer and H_2_O_2_ as an enzyme substrate, in a sandwich-type immunoassay format. Experimental results showed linear relationship between peak current and CEA concentration for the range of 5–60 ng·mL^−1^. The limit of detection was 5 ng·mL^−1^. Several works in the last year have been reported proposing new architectures for the development of sandwich-type graphene-based electrochemical immunosensor for CEA detection, where graphene can acts as sensing platform at electrode surface, label agent for the increase of sensitivity or by performing both activities. Numerous studies in the last year have been reported proposing new architectures for the development of sandwich-type graphene-based electrochemical immunosensor for CEA detection, where graphene can acts as sensing platform at electrode surface, conjugate agent for the increase of sensitivity or perform both activities. The performance of each sandwich-type graphene-based biosensor was compared in Table 1.

**Table 1:** Sandwich-type graphene-based biosensor performance for the detection of carcinoembryonic antigen (CEA).

Electrode	Conjugate	Dynamic Range (ng·mL^−1^)	Detection Limit (ng·mL^−1^)	Ref.
GCE/AuNPs-rGO-Ab1	ZnONPs-rGO-GOD	0.01–80	0.0033	[95]
Graphene/Magnetic Beads-Ab1	AuNPs-horse radish peroxidase (HRP)-Ab2	5–60	5	[97]
rGO/AuNPs-Ab1	Ag/AuNPs-rGO-Ab2	0.0001–200	0.008	[96]
GCE modified with AuNPs-SWCNTs-rGO-Ab1	Pd/Pt-rGO-glucose oxidase (GOD)-Ab2	0.0001–160	3.0 × 10^−5^	[98]
Au electrode/rGO-Nanoporous Au-Ab1	AuNPs/PDDA–GS/MnO_2_-Ab2	0.0003–8	0.08	[99]
Au electrode/AuNps-Ab1	PTC-Arg/Au@rGO complexes	0.001–10	0.0003	[100]
GCE/AuNPs-rGO-hemin-Ab1	AgNPs-rGO–GOD-Ab2	0.0001–160	3.0 × 10^−5^	[101]
rGO-AuNPs modified GCE microfluidic paper analytical devices (μPADs)	P-acid/Pt–Ag Alloy NPs-Ab2	0.001–100	0.0003	[102]

##### Prostate Specific Antigen

Prostate specific antigen (PSA) is a characteristic tumor marker of prostate cancer used in prostate cancer diagnosis and screening. The first attempt to use graphene for sensitive detection of PSA was trough the fabrication of a label-free electrochemical immunosensor. The sensor technology was based on the surface functionalization of GCE electrode with a thin film of the nanocomposite, rGO, 1-pyrenebutanoic acid, succinimidyl ester (PBSE) and CoNPs, as a sensing platform [103]. The PBSE was conjugated with Anti-PSA antibody in order to increase the selectivity for PSA. The measurements suggested that electroactivity of CoNPs was greatly enhanced in the presence of rGO due to its great electron-transfer ability. The specific antibody-antigen immunocomplex formed on the electrode resulted in the decrease of amperometric signal, linearly dependent with PSA concentration in the range of 0.02–2 ng·mL^−1^ with a low detection limit of 0.01 ng·mL^−1^. Recently a new approach based on highly conductive crumpled like structure rGO/AuNPs nanocomposite as a sensing platform for detection of PSA was reported [104]. The 3D immunosensor showed a good linear relationship between the current change and different concentrations of PSA from 0–10 ng·mL^−1^ and the detection limit 0.59 ng·mL^−1^. 

Recently a novel label electrochemical graphene-based immunosensor for PSA was reported. The sensing surface of the GCE was constructed based on the deposition of new amino-functionalized graphene sheet ferrocenecarboxaldehyde composite material (NH2-rGO@FCA) [105]. The sensor label was based on silver hybridized mesoporous silica NPs (Ag@NH_2_-MCM48), able to improve the electron transfer ability of the immunosensor system. The workability of immunosensors system Ag@NH_2_-MCM48/Ab2 and NH_2_-GS@FCA was based on the linear catalytic current increase with the PSA concentration. The immunosensor showed a good relationship between the current responses toward PSA concentration on the range from 0.01 to 10.0 ng·mL^−1^ similar to the non-labeled ones, however the detection limit was as much lower 0.002 ng·mL^−1^. 

##### Carbohydrate Antigen 19-9 and 15-3

Carbohydrate Antigen 19-9 (CA 19-9) is not sensitive or specific to use as a screening test for cancer but can be used as a tumor marker if is produced in abnormal amounts. When CA 19-9 level is elevated to high concentrations it is often correlated to with gastrointestinal malignancies such as cholangiocarcinoma, pancreatic cancer, or colon cancer. Yang *et al*. proposed a novel sandwich-type graphene-based electrochemical immunosensor for the detection of CA19-9 [106]. The sensing surface of GCE was built through the assembly Au-rGO nanocomposite and posterior immobilization of anti-CA19-9, forming the structure Ab1/Au-rGO/GCE. The signal enhancers were designed though the GO surface modification with core shell Au@Pd NPs, thionine (Thi), anti-CA19-9 (Ab2) and Horseradish peroxidase (HRP) forming signal probes Au@Pd-Gra/Thi-Ab2/HRP. During the biorecognition event, the target protein CA19-9 was sandwiched between the primary antibody Ab1/Au-rGO/GCE and the prepared bioconjugates Au@Pd-Gra/Thi-Ab2/HRP, resulting in enhancement of the detectable signal. The results showed that peak currents of the electrochemical immunoassay increased with the increase of CA19-9 concentrations, and exhibited a linear relationship range from 0.015 to 150 U·mL^−1^, with low detection limit of 0.006 U·mL^−1^. 

Carbohydrate Antigen 15-3 (CA 15-3) is a tumor marker used to monitor certain cancers, particularly breast cancer. The first technological approach for the sensitive detection of CA 15-3 using graphene based materials consisted on the development of label-free electrochemical immunosensor [45]. The immunosensor, was designed by the surface modification of GCE with a highly conductive N-doped graphene sheets, that allows a significantly increase of electron transfer and high sensitivity toward CA 15-3. The immunosensor exhibited a low detection limit at 0.012 U/mL and a broad linear response in the range of 0.1–20 U/mL. Recently, it was reported a novel electrochemical graphene based immunosensor for sensitive detection of CA15-3 based on dual signal amplification strategy [107]. For trace tag signal amplifier was synthesized a nanocomposite material based on nanoporous TiO_2_ functionalized with Cd^2+^ for increase the electron transfer rate, followed by the immobilization of the antibody CA15-3 (Ab2), resulting in the following sequence (Ab(2)-f-TiO_2_-Cd^2+^). The GCE sensing platform was developed by immobilization of rGO functionalized with ionic liquid and primary CA15-3 antibody (Ab(1)) resulting in the sequence GS-Ab(1). Through biorecognition reaction, the target protein CA15-3 was sandwiched between the primary antibody GS-Ab1 and the prepared (Ab2-f-TiO_2_-Cd^2+^) bioconjugates, resulting in an enhanced detectable signal. The resultant immumosensor displayed a wide range of linear response (0.02–60 U/mL), ultra-low detection limit (0.008 U/mL), good reproducibility, selectivity and stability towards CA15-3. 

##### Protein p53

**Figure 5:**
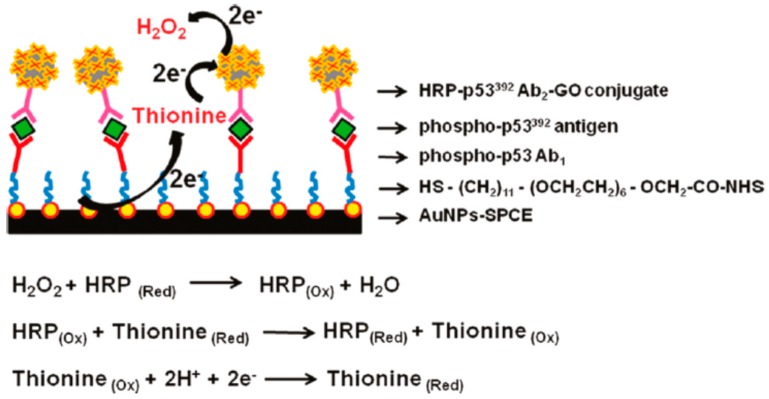
Schematic Illustration of the Multi-enzyme Labeling Amplification Strategy Using HRP-p53392Ab2-GO Conjugate. Reproduced with permission [110]. Copyright 2011, American Chemical Society.

Protein p53 is a well-known tumor suppressor and a transcription factor fundamental in the control of cell growth and modulation of DNA repair processes [108]. Loss of p53 function caused by the conformational changes in p53 protein structure, results in an induction of tumors and gene mutation [109]. Some clinical evidences showed the implication of p53 phosphorylation in human cancers, particularly expression of p53 protein phosphorylated, serine 15 (Ser15) serine 20 (Ser20) and serine 392 (Ser392). In that sense the quantitative analysis of phosphorylated p53 is crucial for early cancer diagnosis. Most of the electrochemical graphene-based immunosensors engineered for sensitive detection of phosphorylated p53, were based on sandwich type strategy. The fist graphene-based immunosensor reported was dedicated to the specific detection of phosphorylated p53 on serine 392. For immunosensor fabrication it was used screen-printed carbon electrode (SPCE) modified with AuNPs to self-assemble a layer of N-hydroxysuccinimideactivated hexa (ethylene glycol) undecane thiol (NHS) for primary attachment of phospho-p53392 capture antibody (Ab1/NHS/AuNPs-SPCE) [110]. The HRP-p53392Ab2-GO conjugate was synthetized by the functionalization of carboxylated GO with HRP and the immobilization of capture antibody p53392 (Ab2) by amidization reaction. After sandwich immunoreaction, the HRP-p53392Ab2-GO captured onto the electrode surface produced an amplified electrocatalytic response by the reduction of enzymatically oxidized thionine in the presence of H_2_O_2_ (Figure 5). The increase of response current was proportional to the phospho-p53392 concentration in the range of 0.02–2 nM with the detection limit of 0.01 nM. 

Another work reported the development of graphene-based immnunnosensor for specific detection of phosphorylated p53 on serine 15. In that case graphene was integrated on the sensing platform instead of the sandwich conjugate platform. For the fabrication of the immunosensor the SPCE was functionalized with graphene and chitosan for primary antibody attachment phospho-p5315 (Ab1/graphene–CHI/SPCE) [111]. The sandwich immunocomplex was formed among phospho-p5315 capture antibody, phosphop5315 antigen, biotinylated phospho-p5315 detection antibody and HRP-labeled streptavidin, resulting in the following system: HRP–streptavidin–biotinAb2/phospho-p5315/Ab1/graphene–CHI/SPCE. The linear response was observed for concentration range of phospho-p5315 from 0.2 to 10 ng·mL^−1^, with the detection limit of 0.1 ng·mL^−1^. 

##### Vascular Endothelial Growth Factor (VEGF)

Vascular endothelial growth factor (VEGF) is an important growth factor that regulates the progression of angiogenesis. VEGF overexpression appears in many human cancers, such as brain tumors, lung cancer, breast cancer, gastrointestinal cancer and urinary tract tumors [112]. Lin *et al*. developed a simple and reusable strategy for detecting the VEGF concentration in human serum using a graphene based biosensor [113]. The sensing platform deposited at Au electrode surface consisted of a nanocomposite of GO-F_3_O_4_NPs functionalized with Avastin, as the specific biorecognition element for VEGF. The amperometric signal was measured by differential pulse voltammetry to quantify the VEGF concentration, being the linear response of the biosensensor observed for the range of VEGF concentrations from 0.03125 to 2 ng·mL^−1^. The biosensors showed a good stability (RSD = 2.36%, n = 50) and a limit of detection of 0.03125 ng·mL^−1^.

##### Alpha-Fetoprotein (AFP)

Alpha-fetoprotein (AFP) is a glycoprotein of fetal component produced during the embryonic period. However, high AFP serum levels can be found mainly in patients with hepatocellular carcinoma or other types of cancer such as, lung cancer, biliary cancer, gastric cancer, pancreatic cancer, teratocarcinoma of the testis. Graphene-based materials have been explored as a sensing platform for the development of noble electrochemical immunoassay in order to amplify the sensitivity on detection of AFP. The pioneer work reported a graphene-based ultrasensitive multiplexed electrochemiluminescent (ECL) immunoassay method for the detection AFP tumor marker [114]. The graphene probe was synthesized by the assembly with poly (diallyldimethylammonium chloride) (PDDA) and luminol-capped gold nanoparticles (PDDA-G@Lu-Au). The obtained PDDA-G@Lu-Au composite particles were used as substrate for antibody and HRP immobilization with high luminol capacity load efficiency. The second probe consisted on multilabel-antibody functionalized core-shell Fe_3_O_4_@Au composites (GMPs). The combination of AFP with the two labels results in sandwich-type immunocomplexe PDDA-G@Lu-Au~HRP-Ab2/AFP/Ab1~GMP. The magnetic sandwich-type immunocomplexes were immobilized on the working screen-printed carbon electrode (SPCEs) by applying a magnetic field. That approach avoids some adverse effects obtained through the surface functionalization of the electrodes. Under the optimized conditions, the ECL method shows a linear range of AFP detection from 0.002 to 20 ng·mL^−1^ and a low detection limit of 0.2 pg·mL^−1^. Recently Zhuo *et al*. proposed the development of a nobel electrochemiluminescent immunosensor based on a new enhancer of peroxydisulfate system for the detection of AFP [100]. The sensor probe design was based on rGO/AuNPs nanocomposites functionalized by supramolecular assembly with arginine covalently bond to 3,4,9,10-perylenetetracarboxylic acid (PTC-Arg/Au@Gra). The PTC-Arg/Au@Gra bioconjugate was used as multifunctional nanocarriers for the absorption of the second antibody of alpha-fetoprotein (Ab2), which formed sandwich-type immunoassay format through association with AFP and the first antibody (Ab1) previously assembled at Au electrode surface. With PTC-Arg/Au@Gra as enhancer of peroxydisulfate system, the immunosensor exhibited a wide dynamic range of 0.001–10 ng·mL^−1^ and detection of limit of 0.3 pg·mL^−1^. 

##### Multiplex Cancer Tumor Markers Detection

Graphene is starting to being explored as an effective platform for electrochemical multiplex detection of actively targeted biomarkers, which has clinical relevance. Recently it was reported the development of a suspended single crystalline graphene (SCG) biosensor for multiplex lung cancer tumor markers detection [115]. After the immobilization process of the antibodies, the label-free suspended SCG sensor was ready for detection of the important lung cancer biomarkers: ANXA2, VEGF, and ENO1. SCG label-free lung cancer biosensor showed a resistive response shift due to the absorption of biomolecules by the bio-receptors on the surface of graphene that cause the change in density and mobility of charge carriers of the film according to different concentrations of the three types of lung cancer tumor markers, ANXA2, VEGF, and ENO1. The biosensor sensitivity studies performed with presence of the three tumor markers showed that the response to the specific antigen is 1 order of magnitude larger than the non-specific ones. The high performance of the SCG biosensor results from synergistic effects obtained from the combination of two important bioevents on its surface: strong interaction between antigen and its bio-receptors at graphene surface and the blocking of the non-specific binding sites on graphene surface by BSA, which further avoids the absorption of non-specific molecules. Furthermore, the suspended structure of SCG biosensor, which has better carrier mobility, sensing area, and low frequency noise is responsible for the enhanced sensitivity with detection limit of 0.1 pg·mL^−1^, good specificity and large linear detection range from 1 pg·mL^−1^ to 1 μg·mL^−1^. This SCG label-free lung cancer biosensor showed to have the lower detection limits ever reported for the three different tumors markers ANXA2, VEGF, and ENO1. 

#### 5.1.2. DNA Biosensors

DNA sensors are common used approach for early cancer diagnosis and mutation detection. Electrochemical sensors are one of most used approaches on DNA technology development, because DNA bases are electroactive which allows direct electrochemical signals recording during the electrooxidation process. Graphene demonstrate superior electrochemical performance for oxidation of DNA bases due to its outstanding electrochemical properties and large surface-to-volume ratio, that play a very important role on the development of new electrodes with increased sensitivity and simultaneous detection [116]. Electrochemical DNA graphene based sensors for low-concentration detection of Breast Cancer 1 (BRCA1) DNA sequences are a very recent technology [117]. It was reported that mutations on this gene were associated with an 80% increased risk of breast and ovarian cancer [118]. The biosensors construct was based on the functionalization of GCE surface with rGO as a sensing platform for the immobilization of DNA capture probe (DNA-c with amino group at 5′ end). In this system, one-half of the DNA target (DNA-t) is allowed to hybridize with the immobilized capture probe (DNA-c), while the other half interacts with the reporter probe (DNA-r) conjugated with gold nanoparticles. Electrochemical oxidation of gold nanoparticle was monitored using cyclic voltammetry to detect the concentration of DNA-t. The results showed that the sensor was stable, reproducible and sensitive and it could detect up to 6 fg·mL^−1^ of DNA target. In another work, Wang *et al*. proposed a novel sensitive and selective electrochemical DNA graphene-based sensor for the detection of BCR/ABL fusion gene in chronic myelogenous leukemia (CML) [42]. CML is a clonal neoplastic disorder of hematopoietic stem cells caused by expression of the chimeric BCR/ABL fusion oncogene (abnormality occurs in more than 95% patients) [119]. The proposed sensor structure consist on the surface functionalization of GCE trough the self-assembly of graphene/chitosan nanocomposite, followed by the electro-polymerization of polyaniline (PANI) and electrodeposition of Au nanoparticles and finally decorated with a functional hairpin structure probe for the detection of BCR/ABL fusion gene of CML. The hybridization event was sensitively transduced to the enzymatically amplified electrochemical current signals, showing a high DNA detection sensitivity and a low detection limit 2.11 pM.

#### 5.1.3. Cell Sensors

**Figure 6:**
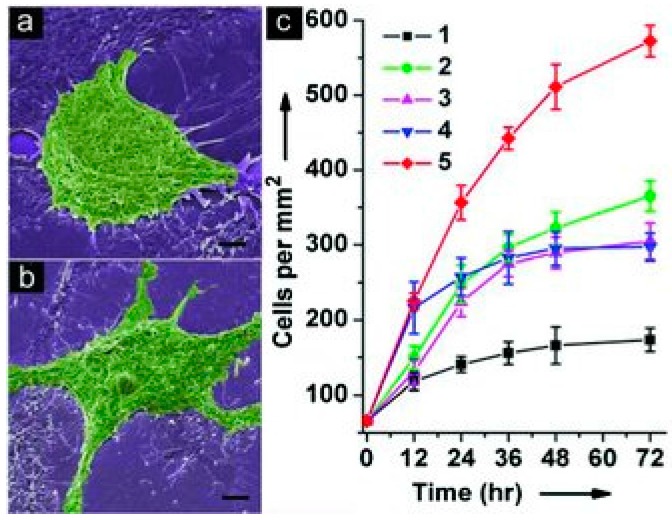
SEM images of MCF-7 cells cultured (12 h) on (**a**) ITO/(AP)_10_ and (**b**) ITO/(graphene-AP)_10_. The scale bar is 5 μm for (**a**) and (**b**). (**c**) Proliferation curves of cells cultured on fi lms with different compositions: 1. ITO/(AP)_10_, 2. ITO/(graphene)_10_, 3. ITO/(graphene–AP)_10_, 4. ITO/(graphene–AP)_10_-laminin (only one layer of laminin on top of the structure), and 5. ITO/(graphene–AP–laminin)_10_. Reproduced with permission [120]. Copyright 2010, WILEY-VCH Verlag GmbH & Co.

Graphene based sensors were also explored for modern diagnosis of cancer by the detection and quantification of specific cancer cells. Graphene based electrochemical sensors has been revealed as the most promising method. Graphene based materials on cells sensors can provide an increase of electrochemical properties in the electrodes and furthermore provides very rich anchor sites to bind recognition species in order to create an interface with high selectivity. The pioneer work of Guo *et al*. reported a smart multilayer amperometric sensor based on deposition of rGO onto an ITO electrode, followed by the functionalization with Prussian blue as H_2_O_2_ catalyst and laminin matrix protein to promote cell adhesion (Figure 6). On this system it was observed that rGO can provide a compatible interface for the growth of human cells and excellent electrical conductivity for electrical detection, allowing for the first time, the *in situ* selective and quantitative extracellular H_2_O_2_ detection. Breast cancer cells were grown on the electrode surface and the sensor was able to detect H_2_O_2_ with a LOD of 0.1 μM upon stimulation [120].

Recently scientists started to give attention to the development new graphene ultrasensitive sensors able to quantify cells that can growth directly on its sensing surface or that can specifically detect cancer cells from a very complex mixture. Wu *et al*. develop a high sensitive graphene based electrochemical sensor through the surface modification of GCE with: chitosan/electrochemically rGO film covalently modified with anti-EpCAM antibodies [121]. This biointerface was then used to detect Hep3B cancer cells. A sandwich system was then generated using CdTe- and ZnSe coated silica nanoparticles that could easily serve as tracing tags to label antiEpCAM and anti-GPC3. Each biorecognition event yields a distinct voltammetric peak, which position and size reflects the corresponding identity and amount of the respective antigen. The combination of ultrasensitive and highly specific electrochemical and fluorescent immunosensing methods showed great potential for application on the detection of circulating tumor cells. The Immunosensor developed exhibited high sensitivity and specificity with excellent stability, reproducibility, and accuracy, with a LOD low then 10 cells·mL^−1^. In another work was reported the preparation of a graphene electrode modified with a new conjugate of peptide nanotubes and folic acid for the selective detection of human cervical cancer cells (HeLa cells) over-expressing folate receptors. The increases number of HeLa cells immobilized at electrode surface promotes a formation of insulating layer inducing the reduction of the recorded intensity of the electrochemical signals. This electrode provides a new sensing alternative for cancer cells detection, with high simplicity related with the synthetic process when compared with the previous one, however it showed higher LOD of 250 cells·mL^−1^ [122]. Feng *et al*. develop a reusable electrochemical graphene-based sensor using aptamer clinical trial II AS1411 for label-free cancer cell detection [123]. Graphene surface was functionalized with 3,4,9,10-perylene tetracarboxylic acid (PTCA) through π–π stacking and hydrophobic interactions in order to introduce more –COOH groups. NH2-modified aptamer strand was linked to PTCA/rGO via covalent bond as the recognition element that can bind to the overexpressed nucleolin on the plasma membrane of cancer cells. The binding of cancer cells decreased the access of the redox probe to the electrode interface. The electrochemical aptasensor showed to be able to differentiate three types of cancer cells (HeLa cells (human cervical carcinoma cell), MDA-MB-231 (human breast cancer cell), K562 cells (leukemia line)) and normal ones (NIH3T3 cells). Another electrochemical graphene-based sensor was developed for specific detection of tumor marker Her2 overexpressed on surface of SKOV-3 tumor cells [124]. The GO modified electrode was covalently functionalized with Anti-Her2 for the capture of SKOV-3 cells, and the unbound HER2 was available to connect with the anti-HER2 graft dsDNA, integrating the advantages of graphene electrochemical properties and DNA marker. This approach provided a very low LOD 5.2 cells·mL^−1^ for SKOV-3 cells. 

Recently, graphene/inorganic nanoparticles hybrids started to be used as a sensing platform at electrodes surface in order to increase the sensitivity of the sensor. Liu *et al.* developed a new ZnO/graphene composite modified with S6 aptamer as sensitive photoelectrochemical (PEC) strategy for the specific detection of SK-BR-3 cancer cells [125]. This hybrid structure was used as sensing platform on ITO working electrode, in order improve its PEC performance. The increase of photocurrent intensity results from the increase in steric hindrances with S6 aptarmer with SK-BR-3 cancer cells receptors. The results showed high selectivity with analogous cells and a LOD of 58 cells·mL^−1^. Yan *et al.* reported a novel graphene immunosensor for detection of cancer cells MCF-7 though the surface modification of the working electrode with GO/AuNPs nanocomposite [126]. The composite was functionalized with aptamer to recognize and bind the tumor marker on the surface of cancer cells MCF-7. Furthermore, thionine functionalized nanoporous PtFe alloy was used as signal amplifier for cells detection. The results obtained showed the increase of the electrochemical signal measured proportional to the concentration of MCF-7 cells. The biosensor showed a good selectivity, acceptable stability and reproducibility with a LOD of 38 cell·mL^−1^. Another work reported the use of small bifunctional composite quantum dot (Fe_3_O_4_/CdSe QD), with intense electrochemiluminescence (ECL) and excellent magnetic property, as reported as enhancer signal probe for sensitive detection of cancer cells via DNA cyclic amplification technique using GO sensing platform to immobilize DNA capture probes (c-DNA1) at the surface of Au electrode [127]. The increased concentration of target cells results in scission of more QDs/DNA signal probe, thus more decrease of ECL signal was obtained. The relationship between the changes of ECL signal and the concentrations of target cells was for the range from 300 to 9000 cells·mL^−1^ with a detection limit of 98 cells·mL^−1^. 

### 5.2. Luminescence Sensors

Graphene as we discuss before was characterized to process very interesting properties, in particular high conductivity and luminescence quenching ability. In fact, the ultra-high quenching efficiency is attributed to three possible mechanisms: Forter resonance energy transfer (FRET), surface energy transfer (SET) and photo-induced electron transfer [41]. As a quencher for diverse luminescence energy donors, graphene materials have been used in combination with several probes.

**Figure 7:**
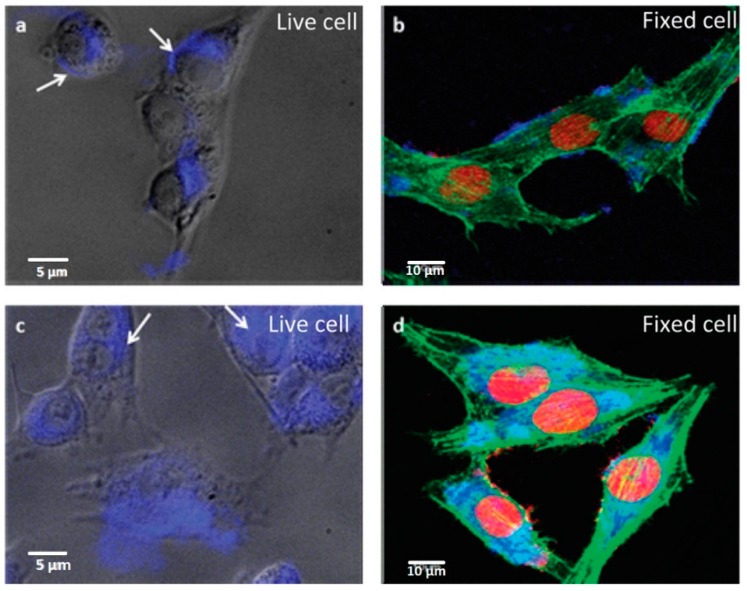
Real-time *in situ* detection of breast cancer cell surface integrin expression by the RGD–pyrene–GO probe: (**a**) probe fluorescence recovery by live MDA-MB-435 cancer cells which overexpress integrin avb3 on the cell surface. The recovered fluorescence is mainly detected on the cell membrane as indicated by white arrows; (**b**) probe fluorescence recovery by MDA-MB-435 cancer cells followed by 4% formalin fixing; (**c**) equivalent concentration of free RGD–pyrene incubated with liveMDA-MB-435 demonstrates significant endocytosis as indicated by white arrows; (**d**) equivalent concentration of RGD–pyrene incubated with MDA-MB-435 followed by 4% formalin fixing. Reproduced with permission [129]. Copyright 2012, Royal Society of Chemistry.

Graphene oxide (GO) based fluorescent biosensor was prepared for real-time *in situ* detection of integrin αvβ3. Integrin have a vital role in cancer cell adhesion, proliferation, migration and metastasis [128]. The biosensor concept is based on the capacity of the sp^2^ aromatic domains of GO to establish noncovalent interactions with pyrene molecules resulting in the quenching effect via fluorescence resonance energy transfer or dipole–dipole coupling effects. On this work GO based biosensor system was initially at a quenching state due to the proximity of RGD–pyrene to GO upon π-π stacking interactions [129]. However, the competitive binding of an RGD receptor, integrin avb3, to the RGD ligand disturbs the adsorption of RGD–pyrene onto the GO surface, resulting in the recovery of pyrene fluorescence with the increasing of concentrations of integrin as illustrated in Figure 7. The biosensors developed showed the capacity for detection of purified integrin protein in buffer and the effectiveness for *in situ* detection of integrin overexpression on cancer cells surface (MDA-MB-435 cell line). 

**Figure 8:**
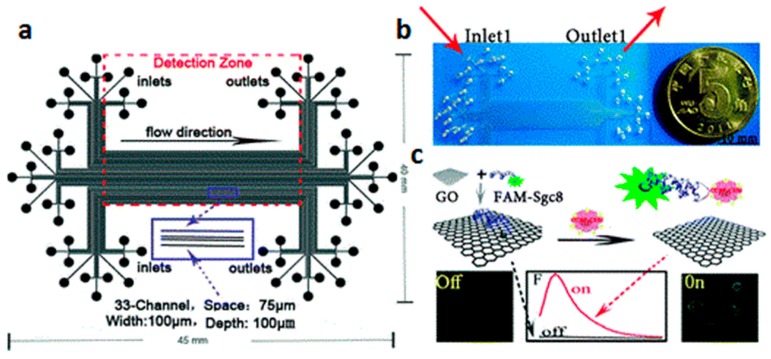
(**a**) Schematic setup and (**b**) the photograph of the GO-based FRET aptasensing microfluidic chip. Scale bar: 10 mm. (**c**) The principle of a ‘signal-on’ aptasensor for detecting CCRF-CEM cells by assaying the cell-induced fluorescence recovery of GO/FAM-Sgc8. Reproduced with permission [130] Copyright 2012, Royal Society of Chemistry.

A multiplex microfluidic chip integrated with the GO-based FRET strategy to create a screening assay for *in situ* detection of tumor cells was also reported [130]. For this purpose, a 33-channel microfluidic chip integrated with the GO-based FRET aptasensor was designed and employed to detect target CCRF-CEM cells with a confocal fluorescence scanning microscope (Figure 8). Initially the FRET probe of GO/FAM-Sgc8 exhibited a quenched fluorescence because of π–π stacking interactions between FAM-Sgc8 and GO. The strong interaction between FAM-Sgc8 and CCRF-CEM cells promotes the release FAM-Sgc8 from GO surface resulting in the recovery of the FAM-Sgc8 fluorescence. The changes observed in fluorescence intensity measurement allowed the quantification of the target cancer cells CCRF-CEM, with the linear response in a concentration range from 2.5 × 10^1^ to 2.5 × 10^4^ cells·mL^−1^ and a detection limit about 25 cells·mL^−1^. Another work suggested a new graphene based fluorescent biosensor system based on a development of a novel molecular aptamer beacon (MA) as fluorescently labelled and GO as the acceptor for target detection by employing long range resonance energy transfer (LrRET) of cellular prion protein (PrPC) [131]. Initially the fluorescence of the designed MAB was completely quenched by GO, however after addition of PrPC the quenched fluorescence was recovered significantly. The results obtained showed that PrPC can be detected over a wide range of 10.2–78.8 µg·mL^−1^ with a detection limit of 0.309 µg·mL^−1^. 

## 6. Conclusions/Outlook

To summarize, we have highlighted the potential of graphene and its derivatives for detection and quantification of cancer biomolecules and cells. Furthermore, we also described numerous procedures capable of convert graphene via surface engineering into specific platforms for particular types of biomolecules or bioreceptors overexpressed by cancerous cells. The biosensing of cancer was discussed giving light to the synergistic properties of the graphene-based sensing entities that can simultaneously act as imaging and therapeutic agents. Additionally, the bioquantification of cancer biomarkers and cells was fully described giving particular attention to specific biomarkers responsible for well-known cancer types.

Although the great potentialities of graphene in biosensing technologies, its use could present some health concern issues since a large number of toxicological studies presented contradictory conclusions [132,133]. *In vitro* and *in vivo* studies have reported that the cytotoxicity of graphene and its derivatives depend on many factors, including preparation protocol, purification processes and final physicochemical properties [30]. Thus, there is a long way to go until these graphene based sensing-systems become optimize and subsequently available for real-world clinical applications. In fact, more detailed studies concerning, for example, the safety of graphene and its derivatives should be addressed with the purpose of monitoring and controlling their metabolic pathway, cellular-uptake mechanism and long term toxicity, which are key-points for nanomaterials applications in bioimaging, drug delivery and cancer therapy. 

The scalable, controllable and reproducible methods of synthesis of graphene-based nanomaterials are other relevant challenges that need to be improved in order to obtain consistent results. 

Overall, cancer is a disease with high innate heterogeneity, very difficult to detect and diagnose via a single biomarker with high specificity and sensitivity. The detection of cancer biomarker in real biological samples such as blood plasma, blood serum cerebrospinal fluid, saliva or urine is a real challenge since these samples are very complex systems with different kinds of proteins, ions and other chemical species able to promote false positive responses in label free assays. Possible ways to overcome this issue are the passivation of the sensor’s surface with an antifouling agent or, more particularly, the previous filtration/purification of the biological sample in order to concentrate the target entity.

Thus, as it was discussed, the ability of graphene based materials to conjugate their outstanding chemical and physical features into remarkable sensing properties appears to be a solid milestone on the long pathway to achieve an early and optimal cancer diagnostics. Indeed, researchers around the world are continuing to explore the wide range of sensing strategies opened by graphene in the last few years due to its proven adaptability and functionality in different cancer microenvironments. For the near future, the ultimate goal is to develop graphene based devices capable of simultaneously detect multiple cancer biomarkers.
